# IgG4‐related mastopathy: A case report and literature review

**DOI:** 10.1002/ccr3.1657

**Published:** 2018-06-22

**Authors:** Takamichi Yokoe, Tetsu Hayashida, Masayuki Kikuchi, Rurina Watanuki, Ayako Nakashoji, Hinako Maeda, Tomoka Toyota, Tomoko Seki, Maiko Takahashi, Eisuke Iwasaki, Shuji Mikami, Kaori Kameyama, Yuko Kitagawa

**Affiliations:** ^1^ Department of Surgery Keio University School of Medicine Tokyo Japan; ^2^ Division of Gastroenterology and Hepatology Department of Internal Medicine Keio University School of Medicine Tokyo Japan; ^3^ Diagnostic Pathology Keio University School of Medicine Tokyo Japan

**Keywords:** breast mass, IgG4‐related disease, IgG4‐related mastopathy, steroid

## Abstract

IgG4‐related sclerosing disease (IgG4‐RD) occasionally involves breast entity, which is often difficult to distinguish from malignant tumor, as both radiologically resembles. We report a case of a breast mass diagnosed as IgG4‐related mastopathy (IgG4‐RM) through needle biopsy, which responded well to glucocorticoid therapy. Unnecessary excision should be avoided.

## 1 INTRODUCTION

IgG4‐related disease (IgG4‐RD) is an increasingly recognized immune‐mediated condition with particular serologic, clinical, and pathological characteristics.^1^, ^2^ Common manifestations include tumor‐like swelling of organs, infiltration of lymphocytes that are enriched in IgG4‐positive plasma cells, and “storiform”‐patterned fibrosis. Furthermore, serum IgG4 levels are elevate in 60%‐70% of patients.^1^ IgG4‐RD that involves the breast has been described in some case reports and small case series, and such cases include IgG4‐related mastopathy (IgG4‐RM), sclerosing mastitis, and inflammatory pseudotumors of the breast. While there are several case reports of IgG4‐related mastopathies,^3^, ^4^, ^5^, ^6^, ^7^, ^8^, ^9^, ^10^ this disease is thought to be very rare, as there have been no reports of large IgG4‐RM case series. IgG4‐RM requires a differential diagnosis from breast cancer, regardless of whether the breast entity is seen in systemic IgG4‐RD setting or in the breast as isolated organ, as the radiological findings of this disease resemble those of a malignant tumor. In addition, IgG4‐RM responds well to glucocorticoid therapy.^5^, ^6^ Few studies, however, have reported the treatment of IgG4‐RM with glucocorticoid therapy, and most cases have undergone excision. Here, we report a case of a breast mass in IgG4‐RD, which was diagnosed as an IgG4‐RM through needle biopsy, and responded well to glucocorticoid therapy.

## 2 CASE HISTORY/EXAMINATION

**Figure 1:**
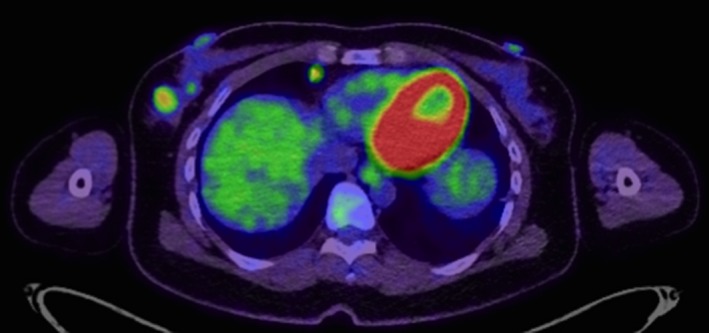
PET‐CT. A right breast mass with high FDG uptake (SUV max 4.97) is shown in the PET‐CT

A 43‐year‐old female consulted her former doctor with a chief complaint of swelling of the bilateral lachrymal and submandibular glands. The laboratory data showed serum IgG4 levels of 515 mg/dL, IgG levels of 2018 mg/dL, IgE (RIST) levels of 230 mg/dL, and negativity for anti‐SS‐A, B antibody. The patient was suspected to be suffering from IgG4‐related sclerosing disease and was introduced to the Department of Rheumatology in our hospital. The positron emission tomography (PET)‐CT scan showed a right breast mass and right axillary lymph nodes with high fluorodeoxyglucose (FDG) uptake (SUVmax 4.97 and 3.08, respectively) (Figure 1), diffuse FDG uptake in the pancreas, and high FDG uptake in the cervix, bilateral lachrymal glands, and submandibular gland. The patient was introduced to our department for the examination of her breast mass, to rule out breast cancer.

## 3 DIFFERENTIAL DIAGNOSIS, INVESTIGATIONS, AND TREATMENT

**Figure 2:**
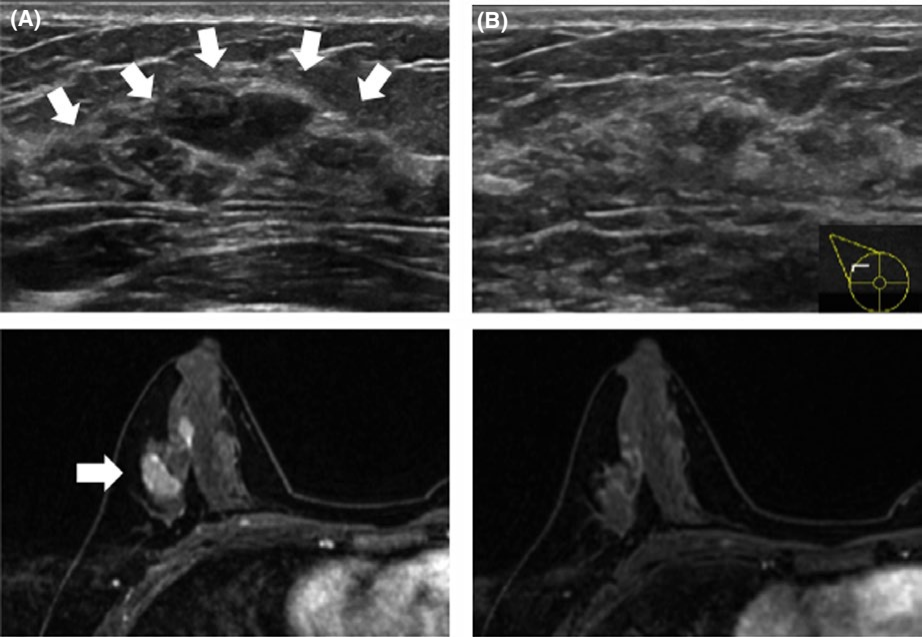
Ultrasonography and MRI of the right breast. A, Before treatment. Ultrasonography showed an irregularly shaped 20 mm low echoic breast mass in the outer‐upper quadrant. MRI noted an irregularly shaped 25 mm contrasted mass. The shape was irregular, the margin was not circumscribed, internal enhancement characteristics were heterogeneous, and kinetic analysis was rapid‐persistent. B, After treatment with 8 weeks of prednisolone. In the ultrasonogram, the right breast low echoic mass almost disappeared. MRI showed the right breast contrasted mass almost disappeared

In the physical examination, the 20 mm‐sized breast mass was palpable, in the right outer‐upper quadrant. The breast ultrasonography showed an irregular‐shaped, 20 mm low echoic breast mass with abundant vascular signal. The breast MRI showed a 25 mm irregular‐shaped contrasted mass (Figure 2). While the shape was irregular, the margin was not circumscribed, the internal enhancement characteristics were heterogeneous, and the kinetic analyses were classified as rapid‐persistent. Thus, the mass was diagnosed as Breast Imaging Reporting and Data System (BI‐RADS) category 4.

**Figure 3:**
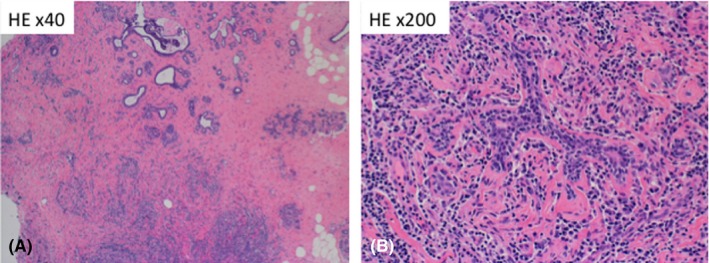
Pathological specimens by vacuum‐assisted biopsy from the right breast mass. A, This micrograph is taken at 40x magnification and stained with hematoxylin and eosin. B, 200x magnification. There was no epithelial neoplasm, but marked lymphoplasmacytic infiltration and dense fibrosis were observed

**Figure 4:**
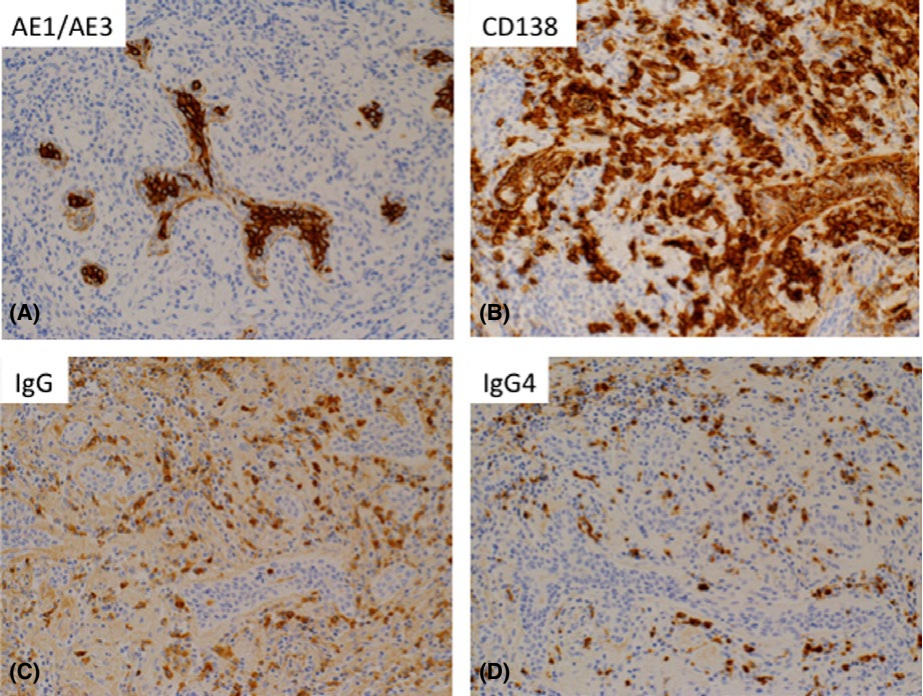
Immunostaining of the pathological specimens by vacuum‐assisted biopsy with (A) cytokeratin (clone: AE1 + AE3), (B) CD138, (C) IgG, and (D) IgG4. Infiltration of numbers of plasma cells is noted. IgG4 was positive in most of the IgG‐positive plasma cells. Tissue IgG4(+)/IgG(+) cell ratio was 85%

Breast cancer cannot be ruled out by radiography alone, and therefore, a vacuum‐assisted biopsy was performed. The pathological findings showed no malignancy, although dense fibrosis and an abundance of plasma cells were observed. By immunostaining for anti‐IgG4 (1:2000, clone HP6025, The Binding Site), IgG4 was positive in 85% of the IgG‐positive cells (Seventy IgG4‐positive cells per high power field) (Figures 3 and 4). Furthermore, the endoscopic ultrasound‐guided fine needle aspiration pathological findings of the pancreas revealed fibrosis with IgG4‐positive cells. From these results, the mass was diagnosed with IgG4‐RM. The cervical cytology showed no evidence of malignancy, and we initiated prednisolone steroid therapy at 30 mg/d, tapered by 5 mg each week.

## 4 OUTCOME AND FOLLOW‐UP

Over the 8 weeks of prednisolone treatment, the breast mass almost disappeared completely under ultrasonography and MRI (Figure 2). We will continue to follow‐up this patient in the future.

## 5 DISCUSSION

The present case showed a breast mass that was diagnosed with IgG4‐related mastopathy (IgG4‐RM) as a part of systemic IgG4‐related sclerosing disease (IgG4‐RD) and responded well to glucocorticoid therapy.

IgG4‐RD can involve one or multiple organs. Multiple organs are affected in 60%‐90% of the patients. Patients of IgG4‐RD often present a mass in the affected organ (eg, orbit, kidney, lung) or diffuse enlargement of an organ (eg, pancreas, salivary glands, submandibular glands, lymph nodes). IgG4‐RD occasionally involves breast entity.

IgG4‐RM or IgG4‐related sclerosing disease of the breast has been described as “sclerosing mastitis” or “inflammatory pseudotumor” in the literature.^3^, ^4^ Cheuk et al^3^ reported four cases of IgG4‐related sclerosing disease of the breast and proposed the descriptive term “IgG4‐related sclerosing mastitis” for such breast lesions, which seem to belong to the syndrome of IgG4‐related sclerosing disease.

It is important to recognize this IgG4‐related entity of the breast, regardless of whether the breast entity is seen in systemic IgG4‐RD setting or in the breast as isolated organ, because the graphical appearance of the entity is similar to that of breast cancer, as in our case.

In the literature, eight articles have reported 12 cases of IgG4‐related mastopathy (Table 1). Most of the patients were female, with ages between 37 and 54 years. The most common symptom was the painless, palpable mass and three patients presented masses in multiple lesions of the breast, while the others had a single lesion. The elevation of serum IgG4 levels was seen in more than half of the patients, and lymphoplasmacyte infiltration with abundant IgG4‐positive plasma cells was seen in the tissue. The breast mass in seven cases manifested as an isolated organ entity, and the breast mass in six cases manifested as a part of systemic IgG4‐RD, which involved eyelid swelling, sialadenitis, lymphadenopathy, or pancreatitis. Excisional diagnostic biopsies were performed in five of the patients, and the other patients were diagnosed with core needle biopsies. All the patients did not experience recurrence, after follow‐ups over a duration of 1‐11 years.

**Table 1:** Case reports of IgG4‐related mastopathy

Case	Sex/age	Symptoms	Lesion/laterality	Serum IgG4 (mg/dL) levels	Tissue IgG4/IgG plasma HPF	Extramanifestations	Diagnosis	Treatment	Outcomes
1^3^	F/48	Painless palpable	Multiple bilateral	350	0.65	N/A	Excision	Excision	No recurrence at 1 y
2^3^	F/51	Painless palpable	Multiple right	3900	0.85	Bilateral eyelid swelling	Excision	Excision	No recurrence at 3 y
3^3^	F/37	Painless palpable	Multiple right	RF 29 IU/L	0.82	Diffuse lymphadenopathy (cervical, axillary, inguinal)	CNB	Observation	Resolution of breast lesion at 6 mo
4^3^	F/54	Painless palpable	Single right	N/A	0.49	N/A	Excision	Excision	No recurrence at 11 y
5^4^	F/46	Induration	Single right	185	N/A	N/A	Excision	Excision	No recurrence at 1 y
6^5^	F/58	N/A	N/A	920	N/A	Mikulicz syndrome AIP	Excision	Excision,PSL	No recurrence at 7 mo
7^6^	F/51	Painless palpable	Single right	217	N/A	Bilateral eyelid swelling	CNB	PSL	No recurrence at 7 mo
8^7^	F/66	Painless palpable	Single left	N/A	0.639	N/A	Excision	N/A	N/A
9^7^	F/45	Painless palpable	Single right	N/A	0.673	N/A	Excision	N/A	N/A
10^8^	F/61	Painless nonpalpable	Single left	RF 122 IU/L	0.5	Chronic sialadenitis, nonalcoholic pancreatitis, cervical mass		N/A	N/A
11^9^	F/52	Asymptomatic	Single left	13.1, IgG 701	N/A	N/A	CNB	Excision	N/A
12^10^	M/48	Palpable	Single right	N/A	0.37, 0.46, 0.51	N/A	CNB	Excision	N/A
Current report	F/43	Palpable	Single right	515, IgG 2018	0.85	Bilateral lachrymal glands and submandibular glands, AIP, cervical mass	VACNB	PSL	No recurrence at 2 mo

F, female; RF, rheumatoid factor; CNB, core needle biopsy; PSL, prednisolone; AIP, autoimmune pancreatitis; VACNB, vacuum‐assisted core needle biopsy.

The diagnostic criteria of IgG4‐RD have not yet been established. Most of the current criteria include: (1) swelling or a mass lesion in one or more organs, (2) elevated level of serum IgG4, and (3) infiltration of the lymphoplasmacytes with abundant IgG4‐positive plasma cells. There have been attempts to establish clear diagnostic criteria since the concept of IgG4‐related disease was proposed in 2001 by Hamano et al.^11^ Recently, comprehensive diagnostic criteria were published from Japan,^12^ and an international consensus has been proposed.^13^

In terms of IgG‐RM, we need histological examination for the diagnosis, as its radiological findings resemble malignant tumor. When we face a case of breast involvement as an isolated organ, IgG4‐RM should be kept in mind as a differential diagnosis, as it is rare. On the other hand, when we face a case of breast involvement as a part of systemic disease, differential diagnosis with malignant tumor is essential, as radiological examination does not provide enough evidence for definitive diagnosis between the two. We have shown in this report that differential diagnosis is possible by the least invasive method: needle biopsy.

In the previously‐reported cases of IgG4‐related sclerosing disease of the breast, half of the patients were treated with excision, rather than with steroid therapy.^3^, ^4^, ^9^, ^10^ With the careful examination of clinical manifestations, serum tests, radiological appearances, and histological examinations with needle biopsy, unnecessary excisional biopsies can be omitted.

IgG4‐RD responds well to steroid therapy.^14^ It is recommended to initiate therapy with prednisolone, at 40 mg/d, and then taper to discontinuation, over 2 months. Responses are evaluated by determining symptom improvements, reductions in the size of the masses, and often, the decrease in serum IgG4 levels. Rituximab is expected to be effective for patients who do not respond to prednisolone at 40 mg/d, who cannot be tapered to under 5 mg/d, or who have strong contraindications to steroid therapy. The treatment of IgG4‐RD also applies to the treatment of IgG‐RM.

## 6 CONCLUSION

In conclusion, IgG4‐related sclerosing disease of the breast is rare. Recognizing this disease is very important, whether it is manifested as a part of systemic IgG4‐RD or as an isolated organ entity, because its clinical and radiological findings are similar to those of malignant tumors. IgG4‐RM can be readily diagnosed with needle biopsy and can be treated with steroid therapy. We would like to highlight that unnecessary surgical biopsy should be avoided.

## CONFLICT OF INTEREST

The authors declare that they have no competing interests.

## AUTHOR'S CONTRIBUTIONS

TY, TH, and EI: interacted with the patient. TY, TH, and EI: identified and acquired relevant reports. SM and KK: diagnosed pathologically. TY: drafted the report. TH, MK, RW, AN, HM, TT, TS, MT, EI, SM, KK, and YK: critically reviewed the report. All authors read and approved the final submitted version.
